# Deer antler stem cells immortalization by modulation of hTERT and the small extracellular vesicles characters

**DOI:** 10.3389/fvets.2024.1440855

**Published:** 2024-10-04

**Authors:** Ze Chen, Deshuang Meng, Xin Pang, Jia Guo, Tiejun Li, Jun Song, Yinghua Peng

**Affiliations:** ^1^Institute of Special Animal and Plant Sciences, Chinese Academy of Agricultural Sciences, Changchun, China; ^2^School of Chemistry and Life Science, Changchun University of Technology, Changchun, China; ^3^Dongfeng Sika Deer Industry Development Service Center, Dongfeng, China

**Keywords:** deer antler stem cell, human telomerase reverse transcriptase, small extracellular vesicles, immortalization, lentivirus

## Abstract

**Background:**

Deer antler stem cells (AnSCs) exhibit properties of both embryonic and mesenchymal stem cells, with superior self-renewal and proliferation, which drive rapid antler growth and regeneration. AnSCs and their derived small extracellular vesicles (sEVs) hold promising potential for applications in regeneration medicine. Due to the restricted proliferative capacity inherent in primary cells, the production capacity of AnSCs and their sEVs are limited. Human telomerase reverse transcriptase (hTERT) is the most important telomerase subunit, hTERT gene insertion has been successfully employed in generating immortalized cell lines.

**Results:**

In this study, we successfully established immortalized AnSCs by transducing the hTERT gene using lentivirus. Compared to primary AnSCs, hTERT-AnSCs demonstrated extended passage potential and accelerated proliferation rates while maintaining the mesenchymal stem cell surface markers CD44 and CD90. Additionally, hTERT-AnSCs retained the capacity for osteogenic, adipogenic, and chondrogenic differentiation. sEVs derived from hTERT-AnSCs exhibited a particle size distribution similar to that of AnSCs, both displaying a cup-shaped morphology and expressing CD81, ALIX, and TSG101, while notably lacking GM130 expression.

**Conclusion:**

We successfully isolated primary stem cells from deer antler and established the immortalized hTERT-AnSCs. Remarkably, this cell line maintains its stem cell characteristics even after 40 passages. The sEVs derived from these cells exhibit identical morphological and structural features to those of primary AnSCs. This research provides essential technical support for the application of AnSCs and their sEVs in regenerative medicine.

## 1 Introduction

Deer antler is the only mammalian organ that can fully regenerate (1), and it has the characteristics of rapid growth (2) and regenerative wound healing (3). Antlers grow at an amazing speed for bones (4), cartilage (5) and nerves (6). The deer antler undergoes physiological regeneration of an organ containing bone, cartilage, blood vessels, nerves, and skin (1, 7, 8). Huge wound will form after the casting of an antler, and these large wounds can heal within 2 weeks, leaving negligible or no traces of scars (9). Rapid antler growth is mainly achieved through the proliferation of cells that reside in the reserve mesenchyme (RM), the outermost layer of the proliferation zone (10). The power of scar-less wound healing on the distal pedicle skin was given by antler stem cell (11). Unlike the regeneration process of salamanders or other animals based on dedifferentiation or redifferentiation, antler regeneration is achieved through general wound healing- and stem cell-based processes (12, 13). Deer antler stem cells (AnSCs) are specialized stem cells found in the mesenchymal tissue of deer antlers. AnSCs not only express mesenchymal stem cell markers such as CD44 and CD90 but also display characteristics reminiscent of those of embryonic stem cells (14). Studying AnSCs provides valuable insights into the mechanisms of tissue regeneration, and this knowledge may have implications for regenerative medicine research and applications in the future (15).

Small extracellular vesicles (sEVs) might generally refer to extracellular vesicles (EVs) <200 nm in diameter, which are cup-shaped spherical vesicles that are released by various cell types, including stem cells (16–18). These sEVs serve as carriers for a diverse range of bioactive substances, including nucleic acids, proteins, lipids, amino acids, and metabolites (19, 20). Notably, their low immunogenicity, minimal toxicity, and precise targeting capabilities make them promising candidates for potential clinical applications in the future (21). Studies have shown that AnSCs and their sEVs have significant effects on the treatment of skin injury, delaying aging and osteoarthritis, and improving postoperative cognitive impairment (22–24). Nevertheless, the limited production capacity of AnSCs and their sEVs is attributed to the constrained proliferative capacity inherent in primary cells (25). The immortalization of AnSCs could be an effective strategy to address this challenge.

Human telomerase reverse transcriptase (hTERT) is an enzyme that plays a crucial role in cellular function, particularly in the maintenance of telomeres (26). hTERT is a catalytic subunit of the telomerase enzyme that can add DNA sequence repeats to the ends of chromosomes, thus preventing or reversing the shortening of telomeres during cell division (27–29). This process is essential for maintaining the replicative capacity and longevity of certain cell types, including stem cells. hTERT gene insertion has been successfully employed in generating immortalized cell lines, including human umbilical cord mesenchymal stem cells (30), horse bone marrow mesenchymal stem cells (31), and dog adipose mesenchymal stem cells (32).

**Figure 1:**
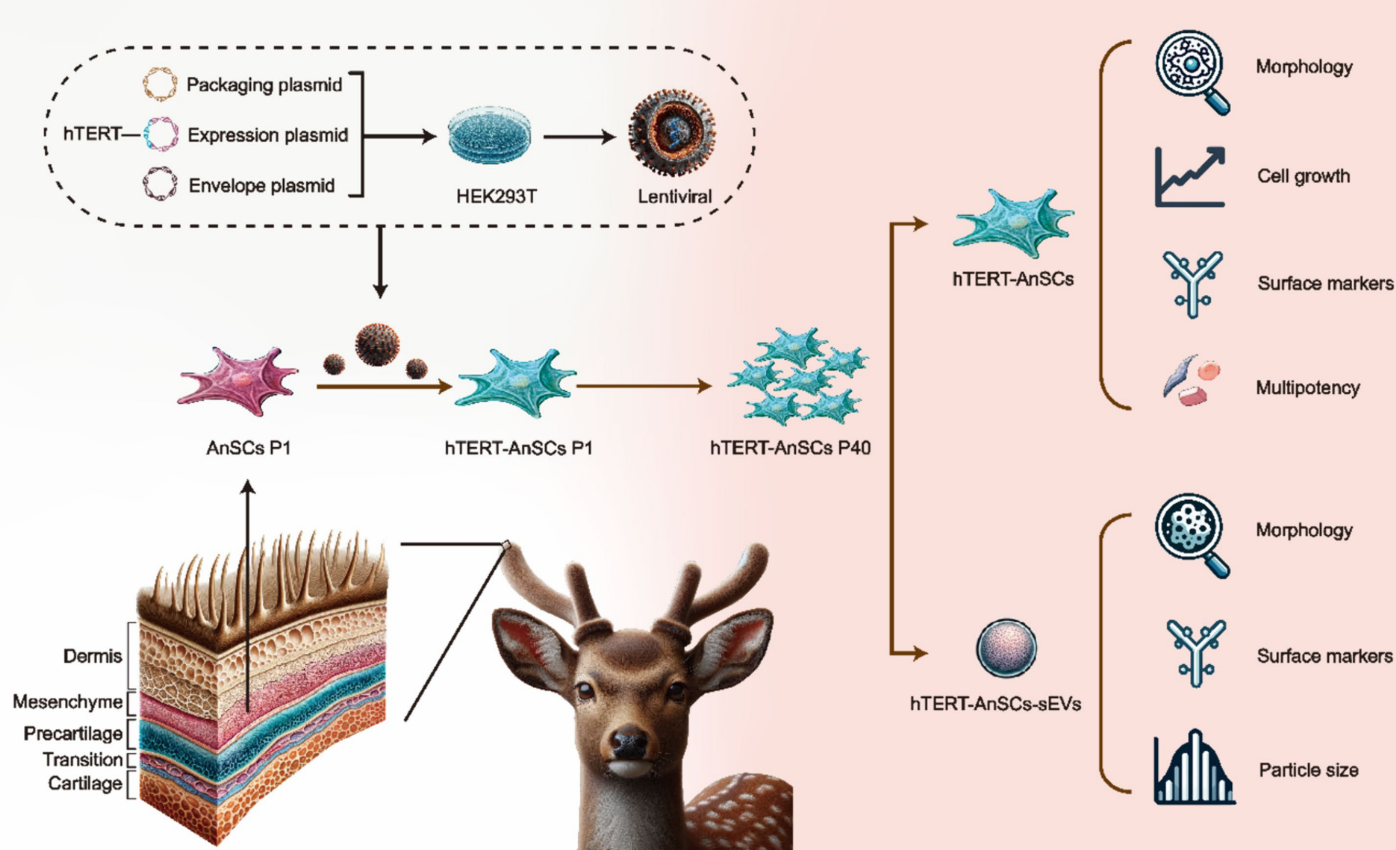
Schematic illustration of the construction of hTERT-AnSCs and characterization of hTERT-AnSCs and hTERT-AnSCs-sEVs.

In this study, we established a lentivirus-based system employing genetic recombination to introduce hTERT into AnSCs, resulting in the development of immortalized AnSCs lines. Subsequently, we focused on determining the preservation of stem cell surface markers and differentiation potential in immortalized AnSCs across multiple generations. Additionally, we examined the morphological characteristics, surface markers, and particle size distribution of sEVs derived from AnSCs before and after the immortalization process (Figure 1). This study represents a groundbreaking achievement in immortalizing antler mesenchymal stem cells through the introduction of hTERT, providing good cell molecular biology experimental material for regenerative medicine research and applications of the sika deer antler stem cell model *in vitro*, and provide the possibility for engineering large-scale production of AnSCs-sEVs.

## 2 Results

### 2.1 hTERT-AnSCs display prolonged cell proliferation potential

**Figure 2:**
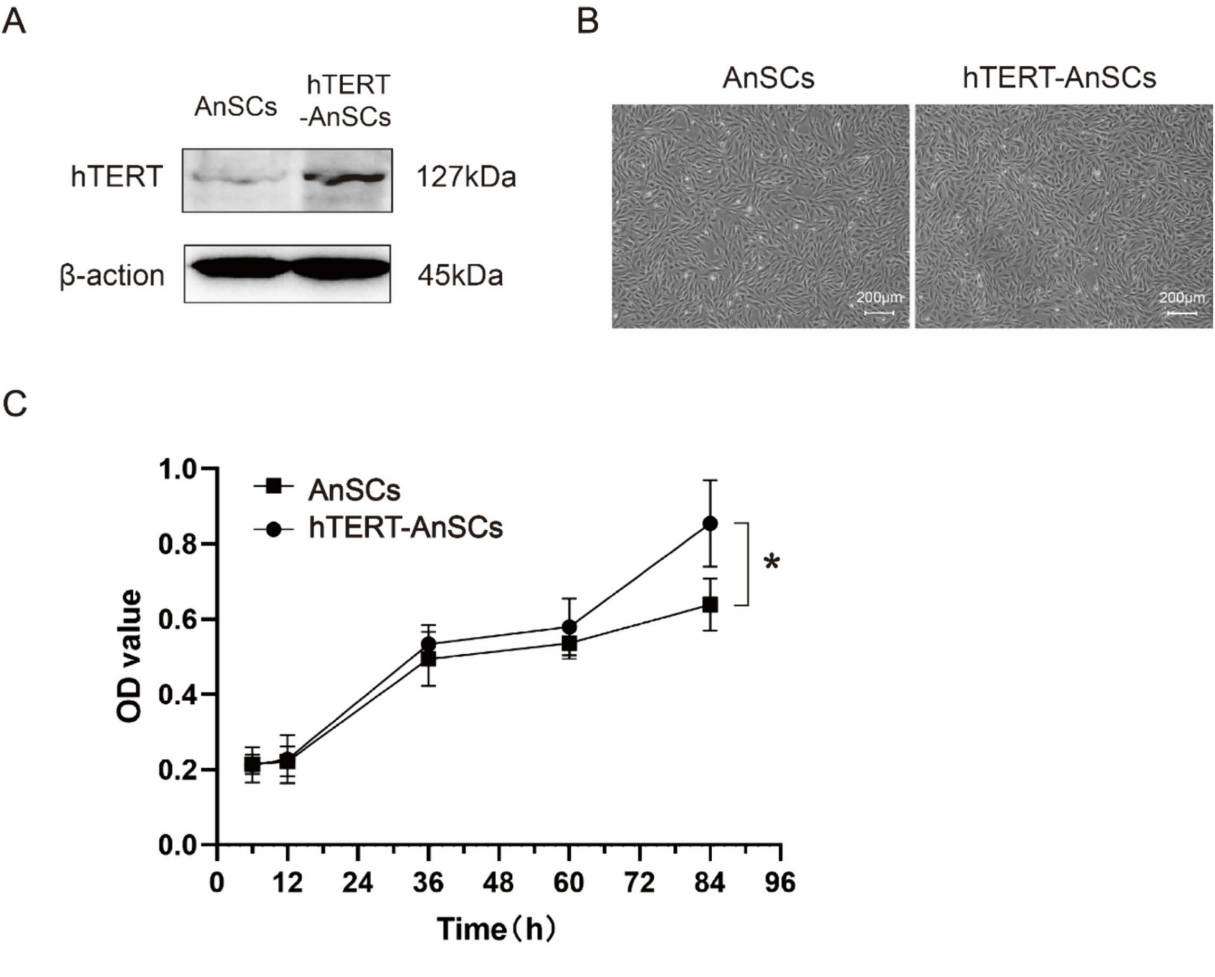
hTERT-AnSCs displayed prolonged cell proliferation potential. **(A)** Immunoblotting was utilized to detect hTERT protein expression in hTERT-AnSCs. **(B)** The cellular morphology and phenotype of hTERT-AnSCs were assessed after 40 passages, while parallel examination was performed on AnSCs after 4 passages for comparison. **(C)** Examination of the cell growth curve throughout successive passages was performed for both AnSCs and hTERT-AnSCs. The average cell number and standard error were determined from triplicate samples.

To generate the PTY-hTERT vector plasmid, the GFP sequence in the PTY-GFP vector plasmid was replaced with the hTERT sequence (Supplementary Figure S1A), while crucial elements such as CMV, IRES, and hygromycin B were preserved (Supplementary Figure S1B). The optimal hygromycin B concentration for selecting hTERT-AnSCs was determined using a screening process with AnSCs, revealing 200 μg/mL hygromycin B as the most effective concentration (Supplementary Figure S2). Subsequently, psPAX2, PMD2. G, and PTY-hTERT were reconstituted into a new lentivirus tri-plasmid system. Following lentivirus production and transduction, hTERT-AnSCs were selected using hygromycin B. The hTERT protein expression level was significantly greater in hTERT-AnSCs than in AnSCs (Figure 2A). Both AnSCs and hTERT-AnSCs displayed a characteristic fibroblastic spindle-like morphology (Figure 2B). A comparison of proliferation abilities between AnSCs and hTERT-AnSCs demonstrated a noteworthy increase in the proliferation rate of hTERT-AnSCs. (Figure 2C). These results suggest that hTERT-AnSCs exhibit an extended potential for cell proliferation.

### 2.2 hTERT-AnSCs maintain the intrinsic immune phenotype

**Figure 3:**
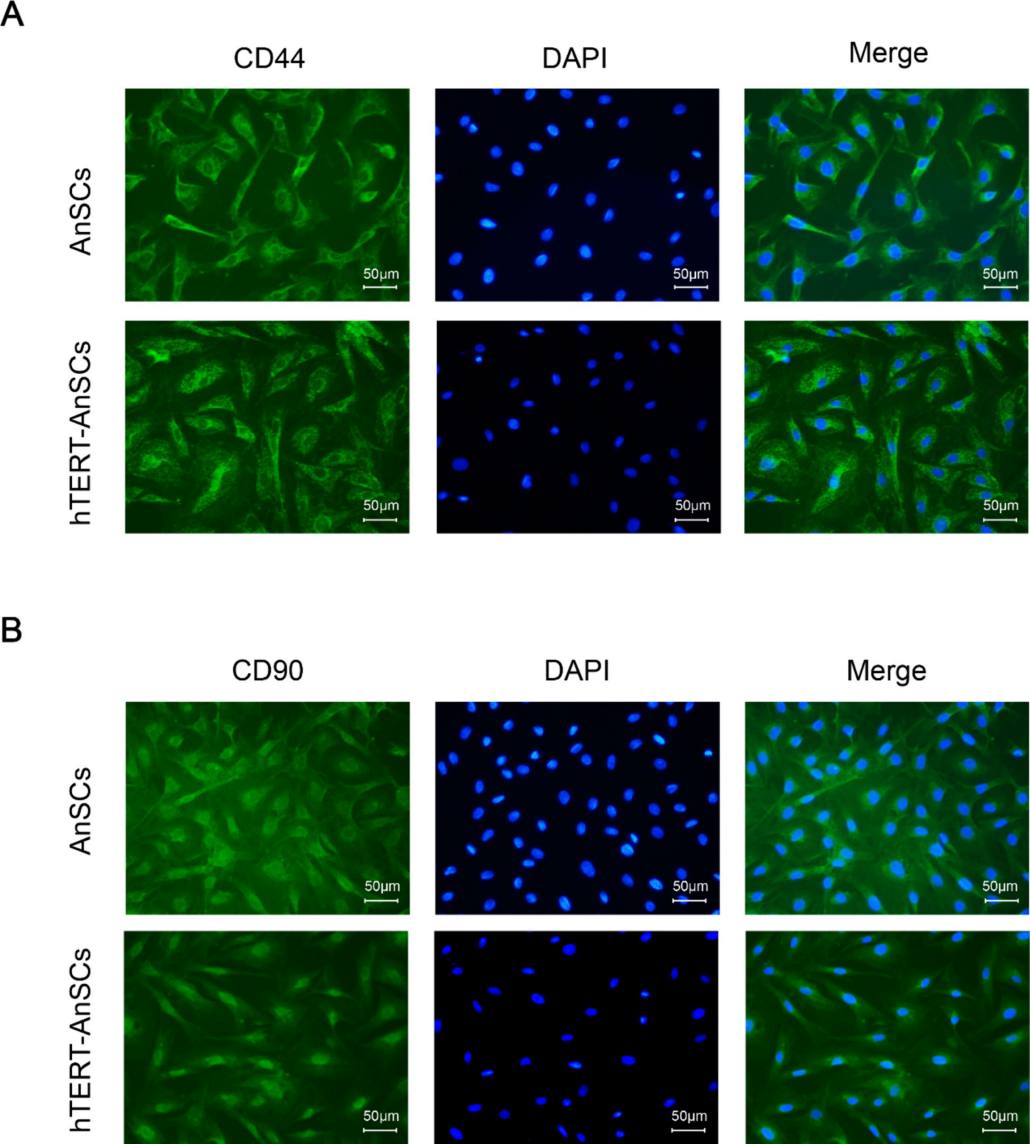
Immunofluorescence showed that hTERT-AnSCs maintained their original immunophenotype. Immunofluorescence staining was performed on AnSCs and hTERT-AnSCs for CD44 **(A)** or CD90 **(B)** (green). Cell nuclei were counterstained with DAPI (blue). Scale bar = 50 μm.

**Figure 4:**
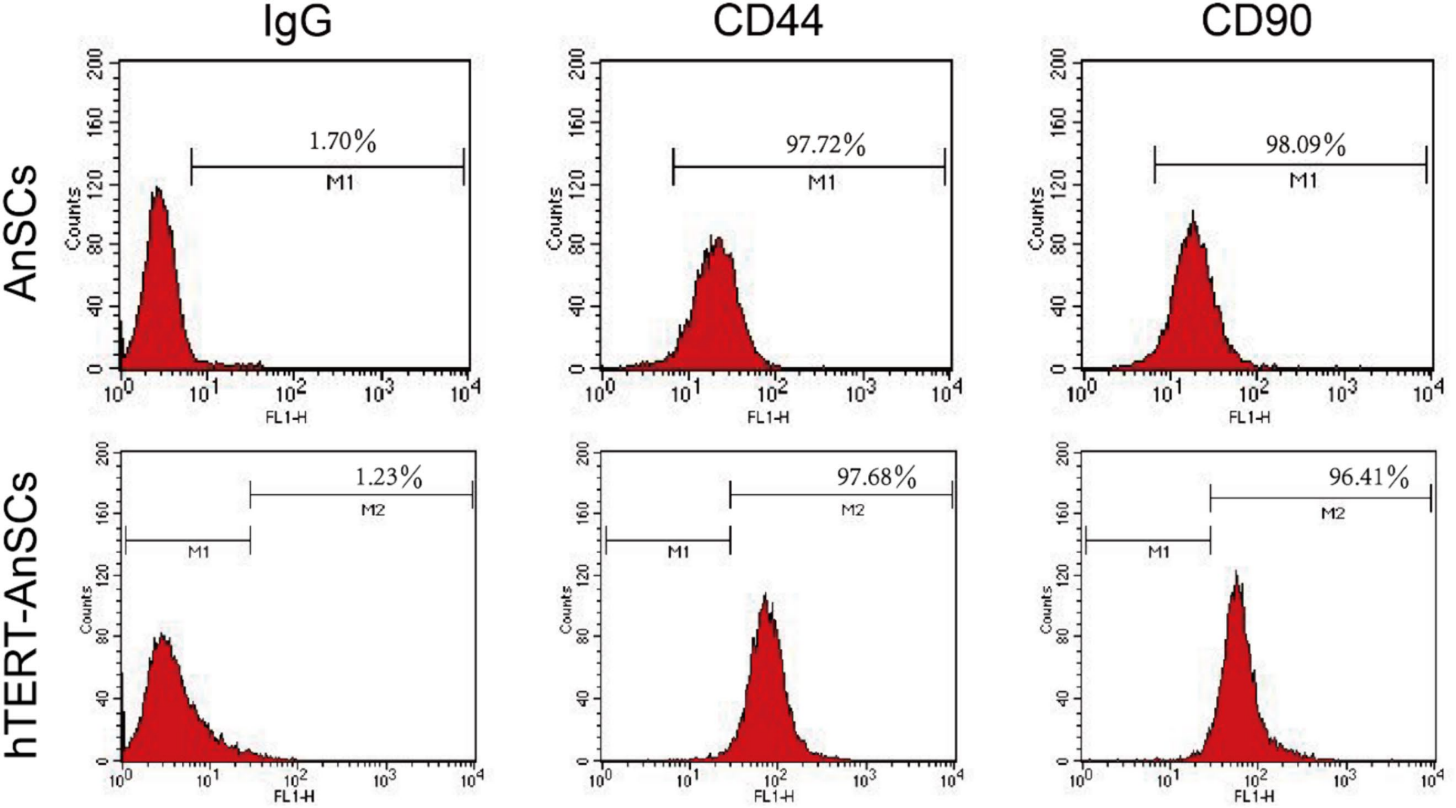
Flow cytometry showed that hTERT-AnSCs retain the intrinsic immunophenotype. Flow cytometry analysis was conducted on AnSCs and hTERT-AnSCs to assess the expression of CD44 and CD90. In the red histograms, the expression of the specified antigens is compared with that of the isotype controls on the left. The values illustrate positive expression patterns of the indicated antigens.

Antler mesenchymal stem cells (AnSCs) constitute a distinct subset of mesenchymal stem cells (MSCs) characterized by the expression of various cell surface markers, including CD44 and CD90. In the identification of hTERT-AnSCs, immunofluorescence showed the positive expression of the mesenchymal marker CD44 in the AnSCs and hTERT-AnSCs (Figure 3A). AnSCs and hTERT-AnSCs also exhibited positive expression of the mesenchymal marker CD90 (Figure 3B). To further examine the immunophenotypes of hTERT-AnSCs, we compared the percentages of CD44- and CD90-positive cells among the two groups of cells using flow cytometry. Flow cytometry analysis revealed positive expression of the mesenchymal markers CD44 (97.72%) and CD90 (98.09%) in AnSCs, and negative for CD34 (0.77%) and CD45 (0.93%) (Supplementary Figure S3). Furthermore, flow cytometry analysis was used to confirm the expression of the mesenchymal markers CD44 (97.68%) and CD90 (96.41%) in hTERT-AnSCs (Figure 4). Together, these results suggest that hTERT-AnSCs exhibit an immune phenotype identical to that of AnSCs.

### 2.3 hTERT-AnSCs maintain multilineage differentiation capacity

**Figure 5:**
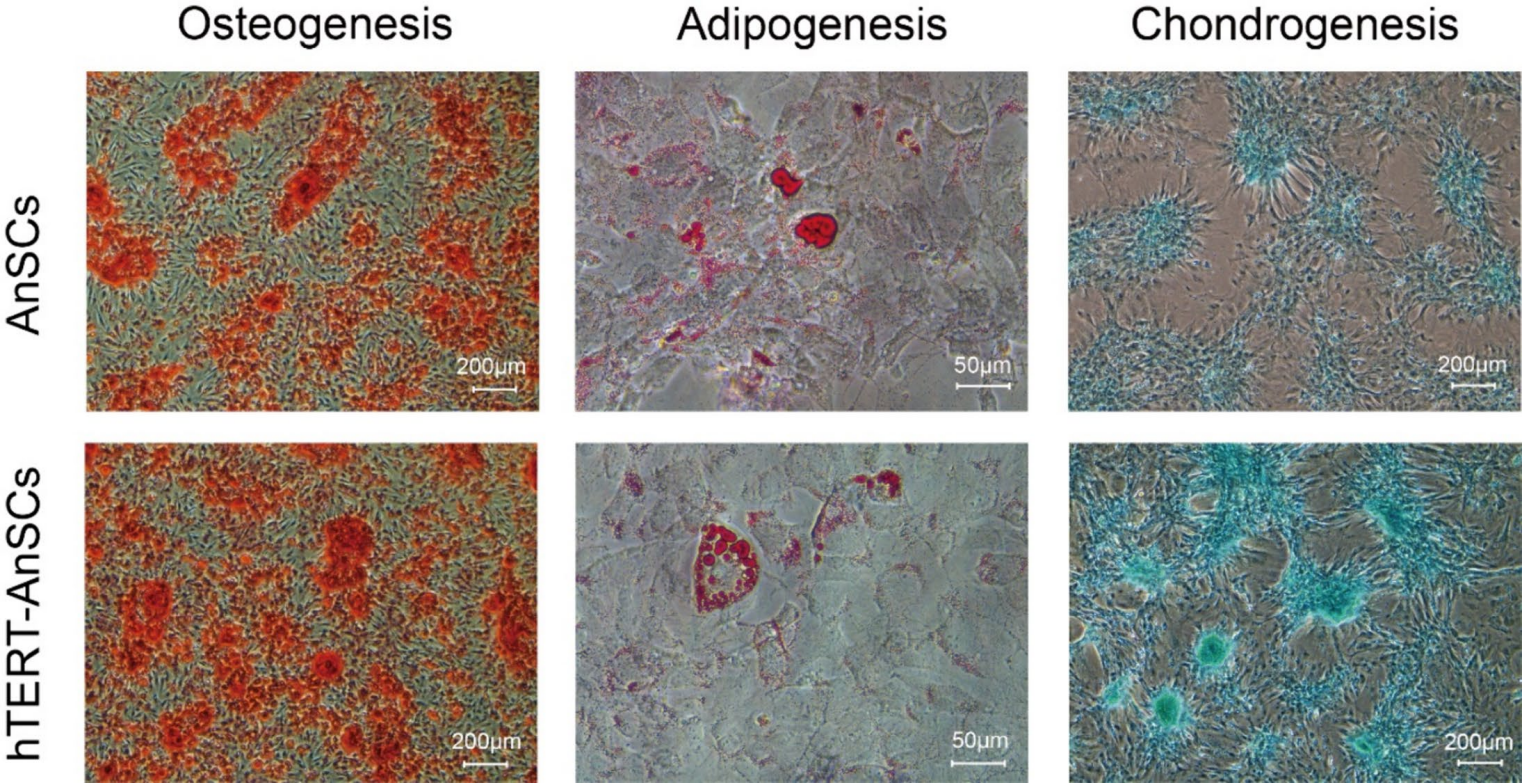
hTERT-AnSCs maintain multilineage differentiation capacity. Upon induction of differentiation, osteocytes (Alizarin Red, scale bar = 200 μm), adipocytes (Oil Red O, scale bar = 50 μm), and chondrocytes (Alcian Blue, scale bar = 200 μm) in both AnSCs and hTERT-AnSCs within the differentiation medium exhibited positive staining.

AnSCs are multipotent cells that can differentiate into various cell types, including osteoblasts, adipocytes, and chondrocytes. Upon osteogenic induction, hTERT-AnSCs exhibited calcified sediment, resulting in a red color after Alizarin Red staining. Similarly, upon induction of lipogenesis, hTERT-AnSCs displayed lipid droplets, which appeared red after Oil Red O staining. Additionally, upon the initiation of chondrogenic induction, hTERT-AnSCs induced the formation of chondrospheres, which were discernible through blue staining after Alcian blue staining (Figure 5). These results indicate that hTERT-AnSCs maintain their original multipotent capability.

### 2.4 hTERT-AnSCs maintain the production and characterization of sEVs

**Figure 6:**
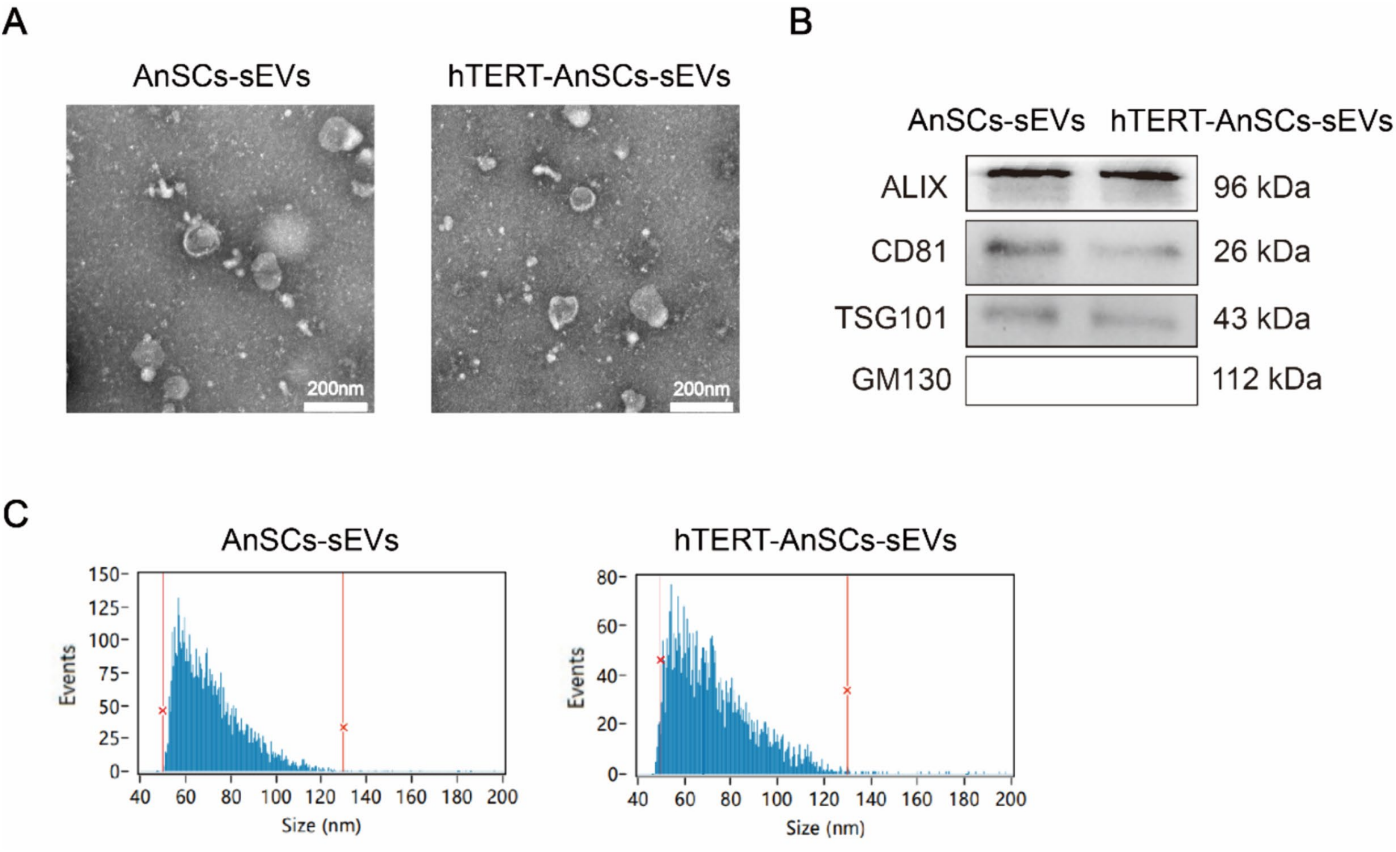
sEVs derived from hTERT-AnSCs retain their original characteristics. **(A)** TEM revealed the morphology of sEVs isolated from both AnSCs and hTERT-AnSCs. **(B)** Western blot analysis of ALIX, CD81, TSG101, and GM130 expression. **(C)** The size of sEVs derived from AnSCs and hTERT-AnSCs was measured using nanoflow cytometry (nFCM).

To evaluate the EV-producing capacity of hTERT-AnSCs and analyse the characteristics of the sEVs derived from these cells. Transmission electron microscopy (TEM) analysis confirmed the presence of a distinct cup-shaped spherical morphology in sEVs isolated from both AnSCs and hTERT-AnSCs, characterized by vesicles with a concave center (Figure 6A). Furthermore, we identified four surface marker proteins (ALIX, CD81, TSG101, and GM130) on these sEVs. Crucially, sEVs derived from both AnSCs and hTERT-AnSCs were positive for ALIX, CD81, and TSG101 but negative for GM130 (Figure 6B). The observed sEVs from both AnSCs and hTERT-AnSCs exhibited a size range spanning from 50 to 130 nm (Figure 6C). These data indicate that the sEVs produced by hTERT-AnSCs are identical to those produced by AnSCs.

## 3 Discussion

A single-cell transcriptomic atlas of antler regeneration revealed the distinct composition of AnSCs, comprising PMC1, PMC2, PMC3, and PMC4 (antler blastema progenitor cells, ABPCs). Notably, PMC4 demonstrates no lipogenic ability (33). This insight led us to select antler tips on the 45th day after casting for AnSCs extraction, as this time point corresponds to a greater number of AnSCs and a lower proportion of PMC4 in the cellular atlas of antler regeneration. Consequently, not all cells in this study that underwent adipogenic differentiation could differentiate into adipocytes, this phenomenon may be caused by the presence of PMC4 cells in AnSCs. But they exhibited a robust capacity to induce osteogenesis and chondrogenesis, this observation aligns with the well-established notion that AnSCs possess a strong propensity for osteogenic and chondrogenic induction.

Deer antler stem cells (AnSCs) exhibit properties of both mesenchymal and embryonic stem cells (ESCs). AnSCs express mesenchymal stem cell markers such as CD29, CD44, CD73, CD90, CD105, CD146, and Stro-1. Additionally, they also express markers such as TERT, nestin, S100A4, nucleostemin, and c-Myc, indicating that they possess certain attributes of ESCs (14). In this study, the mesenchymal stem cell properties were confirmed through immunofluorescence and flow cytometry. The absence of CD34 and CD45 expression in the initial AnSCs indicates that there was no contamination from hematopoietic stem cells. Western blot analysis showed that the TERT gene was expressed in both the initial AnSCs and hTERT-AnSCs. Due to its role in maintaining telomere length, TERT is crucial for the stability of stem cells and cancer cells. TERT is highly expressed in AnSCs, which is expected considering the importance of telomere length in maintaining cell division integrity (14). The exogenous introduction of hTERT extends the lifespan of AnSCs without altering their fundamental properties, such as cell morphology and stem cell characteristics.

The lentivirus introduces the hTERT gene into the cellular genome, leading to its transcription, translation, and the production of the hTERT protein, which functions in the nucleus (34). According to the experimental results of this study, there is no effect on the morphology, surface markers, or particle size distribution of sEVs. Consistent with these findings, previous studies have shown that the morphology, surface markers, and particle size distribution of sEVs did not change significantly before and after the overexpression of Nrf2 in rat bone marrow mesenchymal stem cells via lentivirus (35). Generally, MSC cells can generally propagate for about 8 generations, while hTERT-MSCs can propagate for more than 40 generations. When comparing both cell types with an equal initial number of cells, hTERT-AnSCs yielded more than 1.3 × 10^8^ times more sEVs than AnSCs. This remarkable expansion capacity underscores the potential of hTERT-AnSCs for EV production.

SV40-LT has also been used to establish antler mesenchymal stem cell lines (36). Although SV40-LT can inactivate Rb via p53, promoting cell immortalization by preventing senescence and apoptosis, tumorigenic DNA viruses like simian virus 40 carry significant risks, including the potential for malignant transformation, low efficiency of immortalization, and a high tumorigenic rate (37). Consequently, there are inherent risks associated with the application of sEVs derived from such cells. In this study, the immortalized AnSCs were generated using hTERT, which significantly enhances the safety profile of the sEVs produced.

Although studies have shown that overexpression of hTERT can successfully extend cell lifespan and enhance proliferative capacity, consensus on the long-term stability and safety of these modified cells is still lacking. The potential for tumorigenesis remains controversial, as telomerase activation is a well-known marker of cancer cells. Additionally, different methods of introducing hTERT whether through viral vectors, plasmid transfection, or CRISPR/Cas9 have produced varying results in terms of efficiency and genomic stability. In this study, the construction of hTERT-AnSCs is primarily aimed at generating large quantities of sEVs for the potential application in the beauty industry, where safety concerns are relatively minor. However, further research should focus on its stability, uniformity, and other quality-related factors and functions. Long-term *in vivo* studies are also crucial to evaluate the functional integration and safety of these sEVs, as well as their potential applications in regenerative medicine.

## 4 Conclusion

In summary, this study details the isolation and characterization of AnSCs, followed by the lentivirus-mediated exogenous introduction of hTERT, resulting in the generation of immortalized hTERT-AnSCs. This cell line not only faithfully retains the properties of primary mesenchymal stem cells but also significantly extends their lifespan and enhances their proliferative capacity. Moreover, the sEVs derived from this cell line preserve the morphological and structural features of those derived from primary AnSCs. Immortalized hTERT-AnSCs and their derived sEVs may offer significant potential for applications in regenerative medicine and the beauty industry.

## 5 Materials and methods

### 5.1 AnSCs isolation and culture

The sika deer antler samples were collected from Dongfeng Sika Deer Industry Development Service Center. AnSCs were isolated following an established protocol (32), with RM tissues sourced from 3-year-old male sika deer antler tips immediately following antler cutting during the rapid growth phase (typically 45 days post-casting). RM tissue sample locations were designated based on the work of Li et al. (38). The mesenchymal layer where the RM tissue sample is located is between dermis and permeable. These RM tissue samples were sectioned into small pieces and subjected to digestion in Dulbecco’s modified Eagle’s medium/nutrient mixture F-12 (DMEM/F12) supplemented with 150 units/mL collagenase II, 100 units/mL penicillin, and 100 μg/mL streptomycin (P/S) at 37°C. After digestion, the cells were cultured in DMEM/F12 supplemented with 10% fetal bovine serum (FBS), 100 units/mL penicillin, and 100 μg/mL streptomycin under controlled conditions with 5% CO_2_ at 37°C.

### 5.2 PTY-hTERT lentiviral vector construction

The entire coding region of hTERT was amplified through PCR from the PCI-neo-hTERT plasmid (obtained from the Miao Ling Plasmid Platform) using the primers 5’-ATAAGAATGCGGCCGCG CCACCATG-3′ and 5’-GCCTAGCTAGCTCAGTCCAGGATGG-3′.

DNA clones of hTERT and the PTY-GFP lentiviral vector (gift from Conggang Zhang) were digested with NotI and NheI enzymes (Transgene, China). Following purification via agarose gel electrophoresis, the fragments were ligated using T4 DNA ligase (Transgene, China) to assemble the PTY-hTERT plasmid vector. The recombinant plasmids were transfected into DH5α *E. coli* (Takara, Japan) and subsequently screened for ampicillin resistance. The transformants were screened for correct insertion/orientation of the hTERT fragment by restriction analysis. The positive clones were identified through sequencing by Sangon Biotech Company. The PTY-GFP lentiviral vector without the recombination of hTERT served as the control vector for comparison.

### 5.3 Lentivirus production and transduction

HEK293T cells were seeded in 100 mm plates at a concentration of approximately 5 × 10^5^ cells/mL (10 mL per plate) and maintained at 37°C with 5% CO_2_. After overnight incubation, transfection was carried out using Lipofectamine 3,000 Reagent (Invitrogen, United States) to introduce the packaging plasmid psPAX2, the envelope plasmid pMD2. G, and either PTY-GFP or PTY-hTERT expression plasmids into HEK293T cells. The culture medium was replaced 6 h after transfection. The supernatants containing the virus were collected at both 48 and 72 h post transfection. AnSCs were seeded in 100 mm plates at a density of approximately 5 × 10^5^ cells/mL (10 mL per plate) and then incubated at 37°C with 5% CO_2_. After filtration, the virus-containing supernatants were added to the AnSCs in the presence of 8 μg/mL polybrene. For cell screening, hygromycin B was applied to both hTERT-AnSCs and GFP-AnSCs.

### 5.4 Cell morphology

To evaluate the preservation of normal phenotypic features across passages, the morphological changes of hTERT-AnSCs were compared with those of AnSCs using a ECLIPSE Ts2 FL/Ts2 microscope (Nikon, Japan).

### 5.5 Western blotting

hTERT-AnSCs and AnSCs were seeded at a density of 1 × 10^5^ cells per well in 6-well plates and incubated for 24 h with 5% CO_2_ at 37°C. Total proteins were extracted using RIPA lysis buffer (Beyotime, China), and equal amounts were loaded for each cell extract. Proteins from these extracts were separated via 8% SDS–PAGE and subsequently transferred to PVDF membranes. The membranes were blocked using 5% nonfat dry milk and then incubated with the corresponding primary antibodies at 4°C overnight. After three washes with 0.05% TBST (pH = 7.4), the membranes were incubated with secondary antibody-HRP conjugates for 1 h at room temperature. The resulting bands were visualized using an enhanced chemiluminescence (ECL) detection reagent (Thermo Scientific, United States) and a multi-chemiluminescence image analysis system (Tanon, China). All antibody information used is shown in Supplementary Table S1.

### 5.6 Cell growth curves

hTERT-AnSCs and AnSCs were seeded in five 96-well plates, each containing five replicate wells. The cells were plated at a concentration of 7 × 10^4^ cells per milliliter (100 μL per well) and incubated at 37°C with 5% CO_2_. The viability of both hTERT-AnSCs and AnSCs was evaluated using a CCK-8 cell proliferation detection kit. (Keygen, China). CCK-8 assays were performed at various time points: 6 h, 12 h, 36 h, 60 h and 84 h. The optical density of each well was measured using a microplate reader (Bio-Tek, United States).

### 5.7 Immunofluorescence

hTERT-AnSCs were seeded in 12-well plates at a density of 1 × 10^5^ cells per well and incubated for 24 h at 37°C with 5% CO_2_. After removing the supernatant, the cells were fixed in 4% paraformaldehyde at room temperature for 30 min. Subsequently, the cells were washed three times with PBS and then blocked with 5% bovine serum albumin for 30 min. CD90 and CD44 antibodies were incubated with the samples for 18 h at 4°C. After washing with PBS, the cells were incubated with a goat polyclonal secondary antibody against rabbit IgG-H&L for 1 h. Furthermore, 5 min of counterstaining with DAPI was performed. Cell evaluation was performed using a fluorescence microscope (Nikon, Japan) before another round of three PBS washes.

### 5.8 Flow cytometry

hTERT-AnSCs and AnSCs were plated in 100 mm culture dishes at a density of 1 × 10^6^ cells per dish and incubated for 24 h at 37°C with 5% CO_2_. After being harvested with trypsin, the cells were blocked with 5% bovine serum albumin. Subsequently, the cells were incubated with CD44, CD90, CD34, CD45 and IgG antibodies for 1 h at room temperature, followed by washing with cold PBS. Anti-IgG (H + L) Fluor488-conjugated antibodies were applied for 30 min at room temperature. After another cold PBS wash, the cells were analysed using a BD FACSCalibur flow cytometer (BD Biosciences, United States), with a minimum of 10,000 events collected for each sample. The results were analysed using BD CellQuest Pro software (BD Biosciences, United States). Histogram markers were set based on the negative controls, and the percentage of positive cells was determined using histogram statistics.

### 5.9 Multipotency

The differentiation capacity of hTERT-AnSCs and AnSCs was assessed through osteogenic, lipogenic, and chondrogenic induction. For osteogenic differentiation, cells were seeded into 6-well culture plates at a density of 1 × 10^4^ cells/cm^2^ and cultured in osteogenic differentiation medium consisting of DMEM, 10% FBS, 1% P/S, 50 μg/mL ascorbic acid, 10 mM *β*-glycerophosphate, and 100 nM dexamethasone for a period of 2 weeks. Osteogenic potential was assessed using the Alizarin Red S Staining Kit for Osteogenesis (Beyotime, China). For adipogenic differentiation, cells were placed into 6-well culture plates at a density of 1 × 10^4^ cells/cm^2^ and cultured in adipogenic differentiation medium. The medium included DMEM, 10% FBS, 1% P/S, 0.5 mM IBMX, 5 μg/mL insulin, 0.25 μM dexamethasone, and 1 μM rosiglitazone, and induction was carried out for a period of 2 weeks. Adipogenic potential was assessed through staining with the Modified Oil Red O Staining Kit (Beyotime, China). For chondrogenic differentiation, cells were seeded into 6-well culture plates at a density of 1 × 10^4^ cells/cm^2^ and cultured in chondrogenic differentiation medium (comprising DMEM, 10% FBS, 1% P/S, 10 ng/mL TGF-β1, 6.25 μg/mL insulin, 0.1 μM dexamethasone, and 50 μM ascorbic acid) for 1 week. Chondrogenic potential was assessed using an Alcian blue stain kit (Solarbio, China).

### 5.10 sEVs isolation and identification

sEVs were isolated and characterized following established protocols (23) (39). The removal of cells was achieved through centrifugation at 300 × g for 10 min, while dead cells were eliminated by centrifugation at 2000 × g for 30 min. Further separation of the cell debris was performed through centrifugation at 10000 × g for 30 min. Subsequently, the supernatant was filtered through a 0.22 μm pore size filter to eliminate larger particles. To ensure the removal of aggregates of biopolymers, apoptotic bodies, and other structures denser than sEVs, the solution was centrifuged at 120000 × g for 120 min using a BECKMAN COULTER Optima XPN-100 Ultracentrifuge-SW 40 Ti (Beckman, United States). The sEVs were subsequently collected in 200 μL of PBS and stored at −80°C for future experiments. Transmission electron microscopy (TEM) (Philips, Netherlands) was utilized to evaluate the morphology of the sEVs. Nanoflow cytometry (nFCM) (NanoFCM, China) was used to assess the size distribution and concentration of the particles.

### 5.11 Statistical methods

All the data are representative of three or more independent experiments and are expressed as the mean ± SEM. To compare two groups, Student’s t test was conducted using GraphPad Prism version 9.0. Statistical significance was indicated as follows: *p < 0.05.

## Data Availability

The original contributions presented in the study are included in the article/Supplementary material, further inquiries can be directed to the corresponding author/s.
