# Supplementary material for: Deer antler stem cells immortalization by modulation of hTERT and the small extracellular vesicles characters
Source: Front Vet Sci. 2024 Oct 4;11:1440855. doi: 10.3389/fvets.2024.1440855 (PMC11486761; doi:10.3389/fvets.2024.1440855)
Supplement: Supplementary file 1 [file Presentation_1.PPTX]

## Slide 1
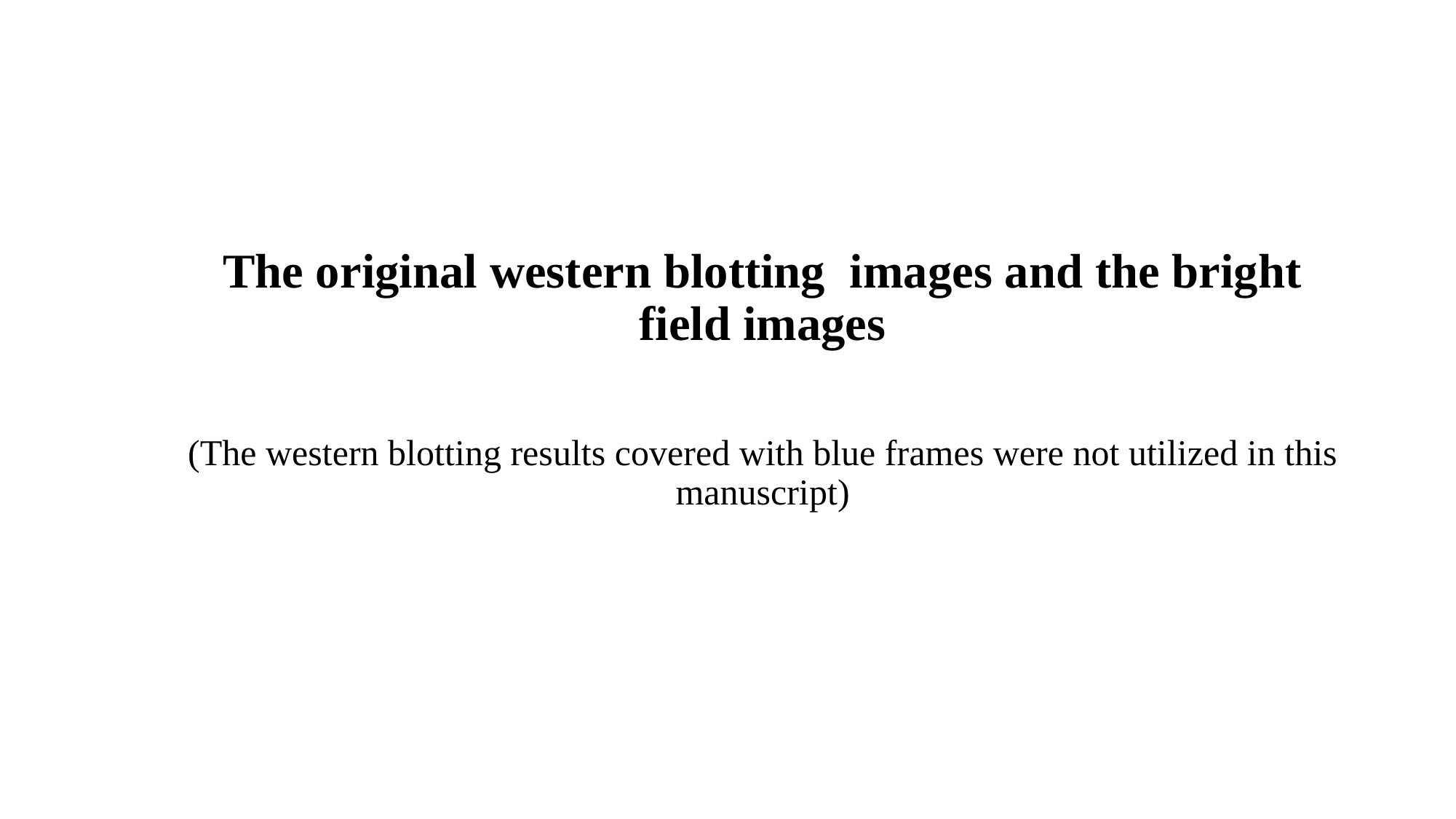

# The original western blotting images and the bright field images
(The western blotting results covered with blue frames were not utilized in this manuscript)

## Slide 2
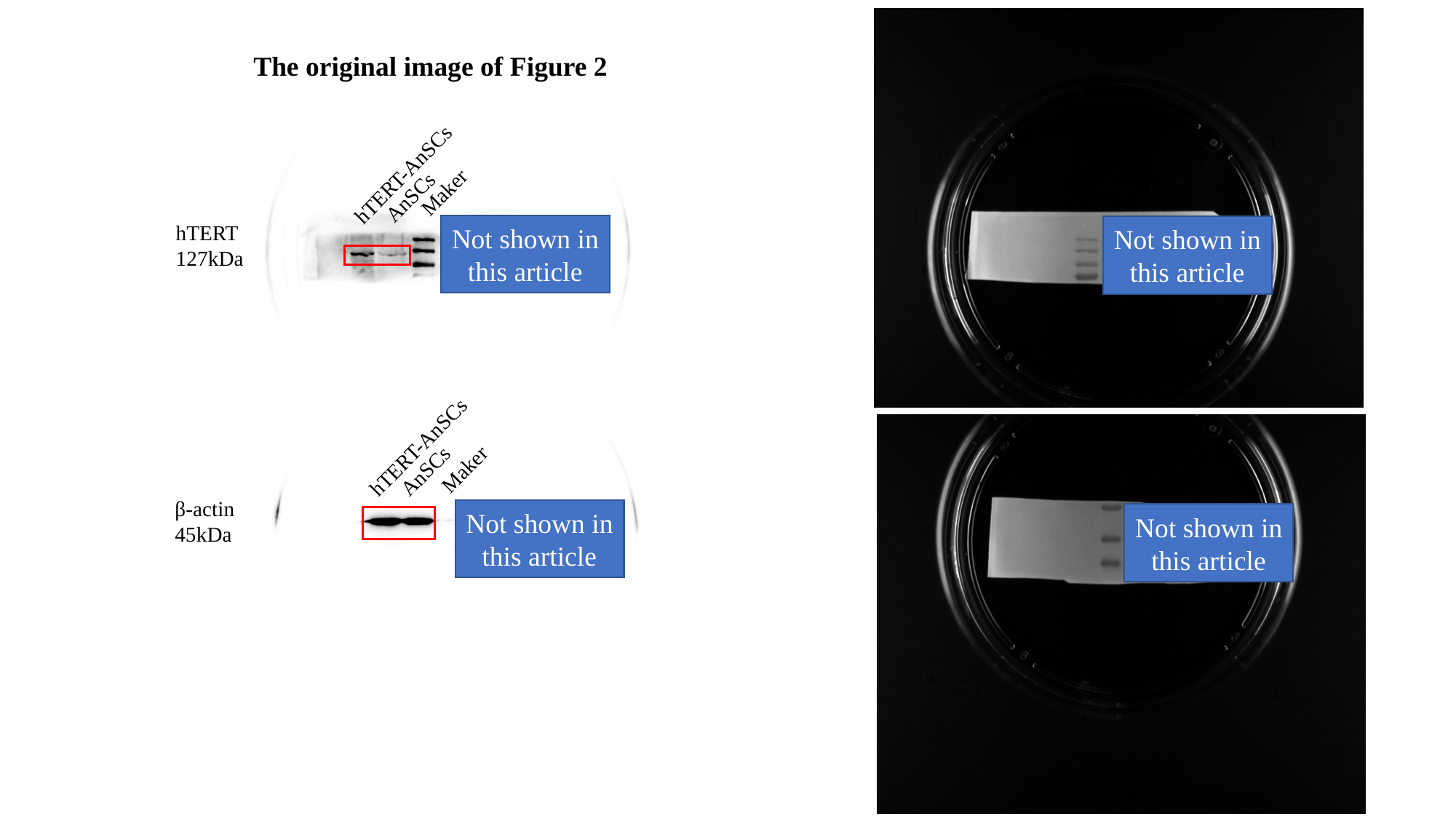

The original image of Figure 2
hTERT-AnSCs
Maker
AnSCs
hTERT
127kDa
Not shown in this article
Not shown in this article
hTERT-AnSCs
Maker
AnSCs
β-actin
45kDa
Not shown in this article
Not shown in this article

## Slide 3
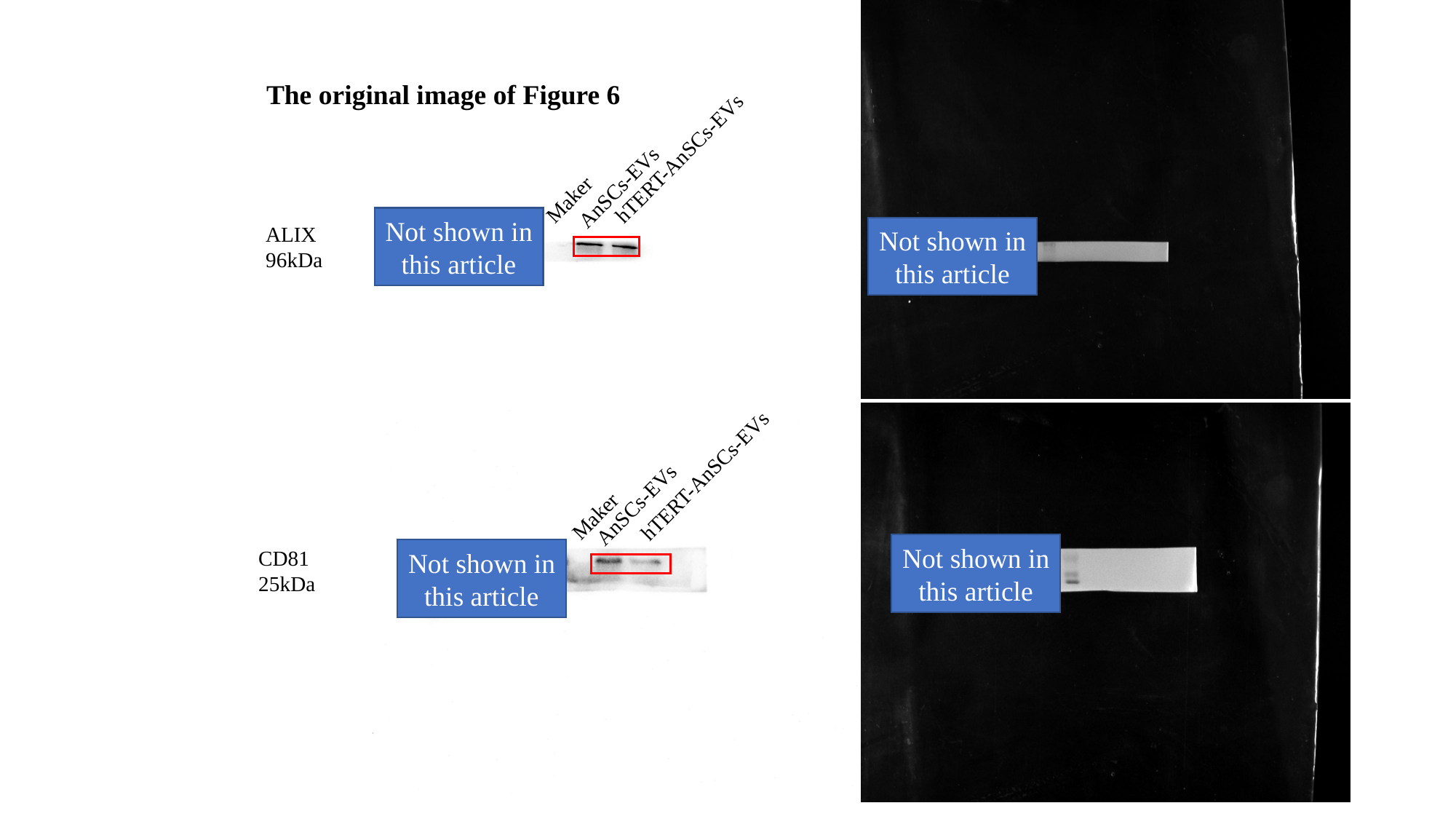

The original image of Figure 6
hTERT-AnSCs-EVs
AnSCs-EVs
Maker
Not shown in this article
ALIX
96kDa
Not shown in this article
hTERT-AnSCs-EVs
AnSCs-EVs
Maker
Not shown in this article
CD81
25kDa
Not shown in this article

## Slide 4
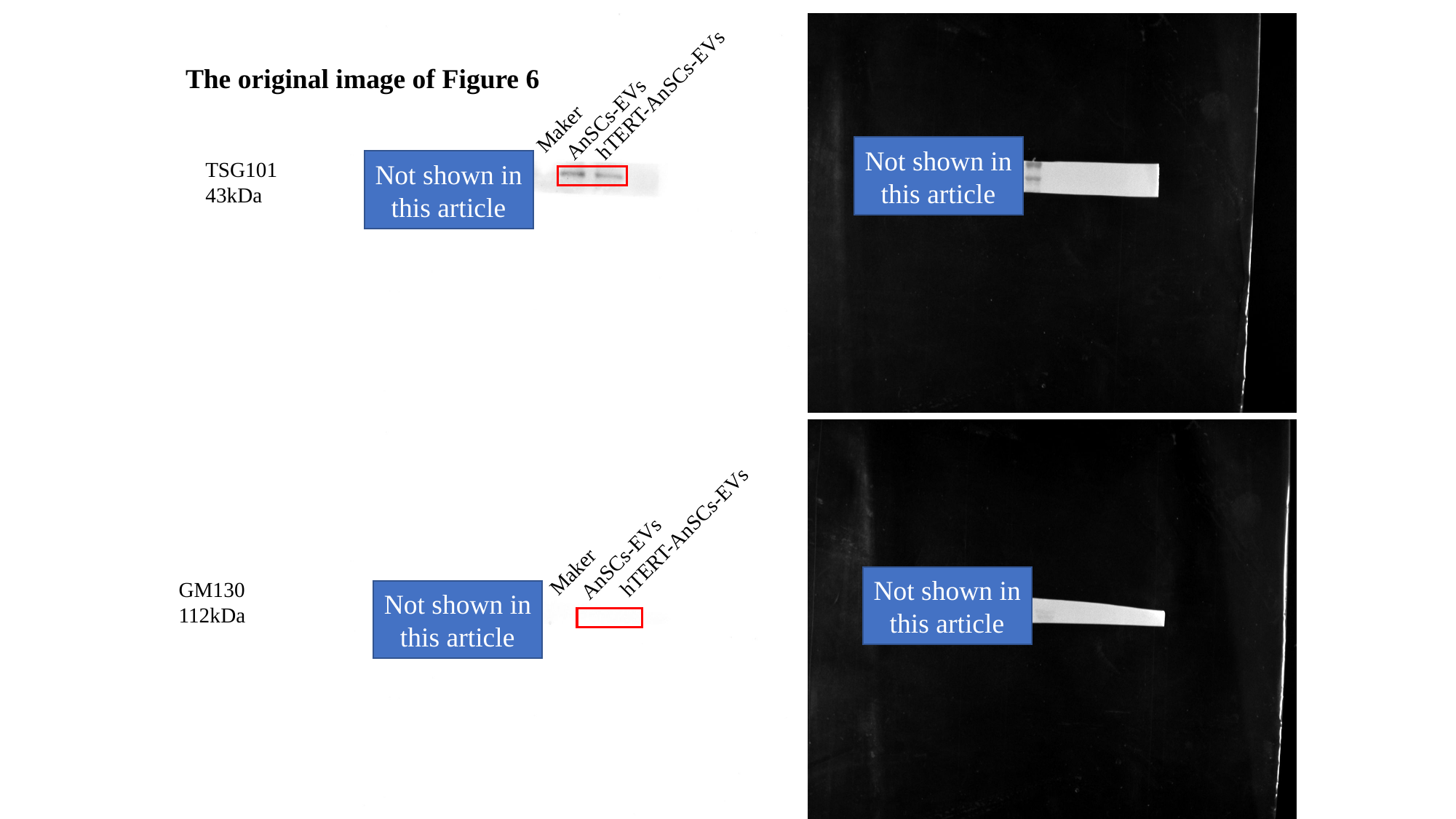

The original image of Figure 6
hTERT-AnSCs-EVs
AnSCs-EVs
Maker
Not shown in this article
TSG101
43kDa
Not shown in this article
hTERT-AnSCs-EVs
AnSCs-EVs
Maker
Not shown in this article
GM130
112kDa
Not shown in this article
