# Supplementary material for: Deer antler stem cells immortalization by modulation of hTERT and the small extracellular vesicles characters
Source: Front Vet Sci. 2024 Oct 4;11:1440855. doi: 10.3389/fvets.2024.1440855 (PMC11486761; doi:10.3389/fvets.2024.1440855)
Supplement: Supplementary file 2 [file Table_1.DOCX]

**SUPPLEMENTARY MATERIALS**


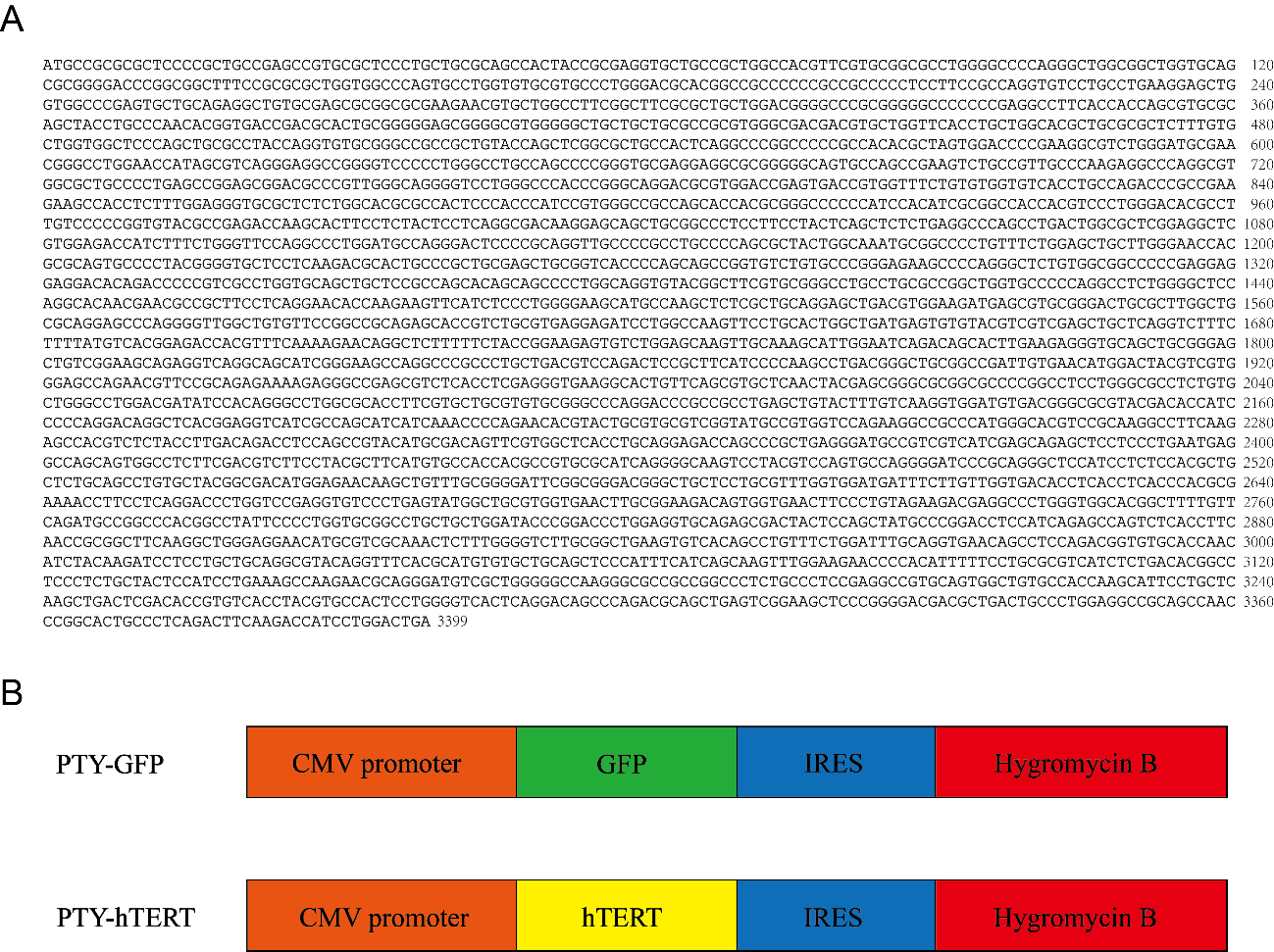


**Figure S1. The sequence of the hTERT gene and construction of the recombinant vector PTY-hTERT.** (A) The cDNA for the hTERT gene comprises 3399 base pairs of nucleotides. The numbers on the right refer to the positions of the nucleotides. (B) Partial diagrams of the PTY-GFP vector plasmid and PTY-hTERT vector plasmid.


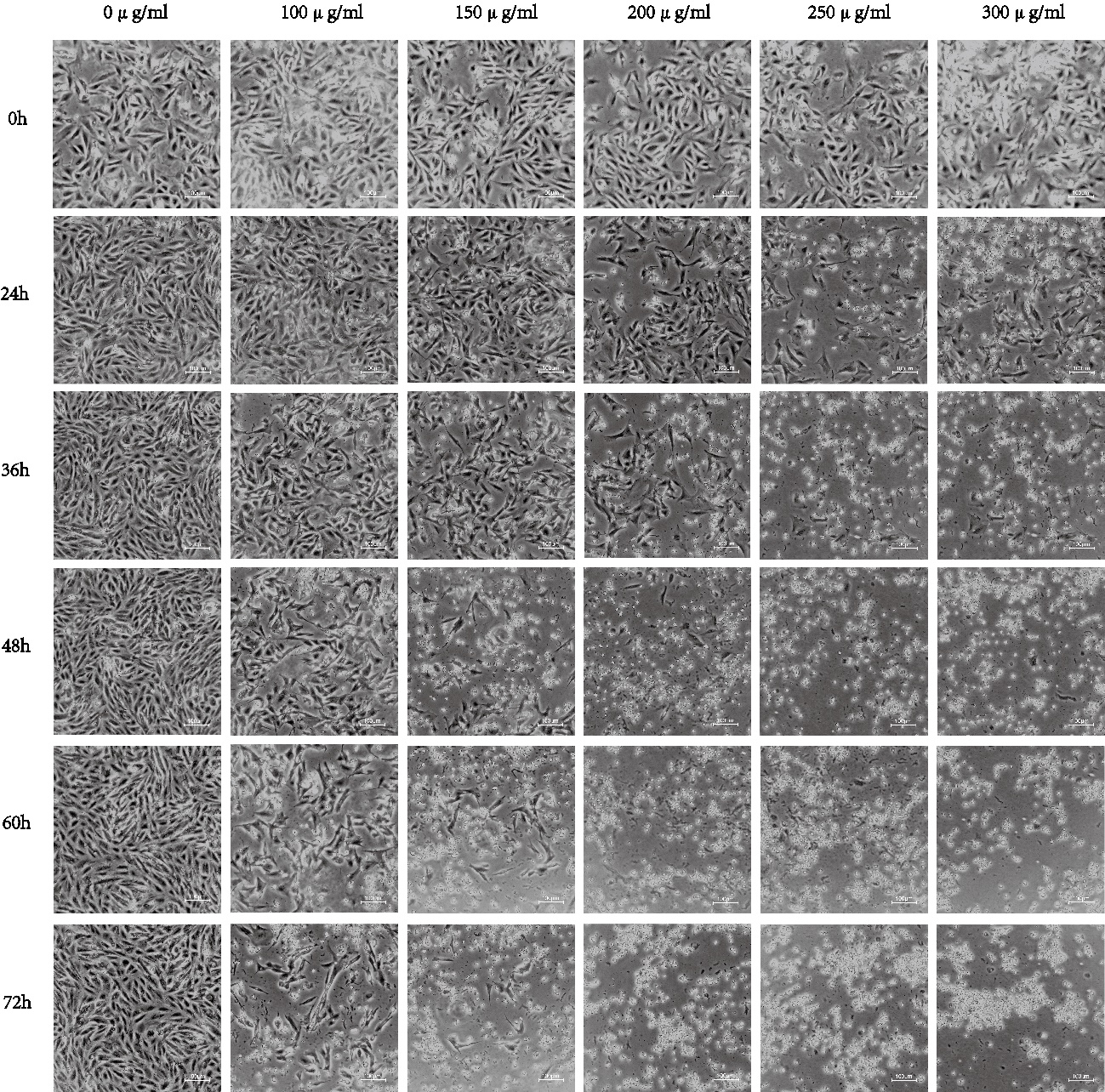


**Figure S2. Determination of the optimal hygromycin concentration.** The effects of hygromycin on AnSCs were examined at various concentrations (0, 100, 150, 200, 250, and 300 μg/ml) at 0, 24, 36, 48, 60, and 72 h, respectively. Scale bar = 100 µm.


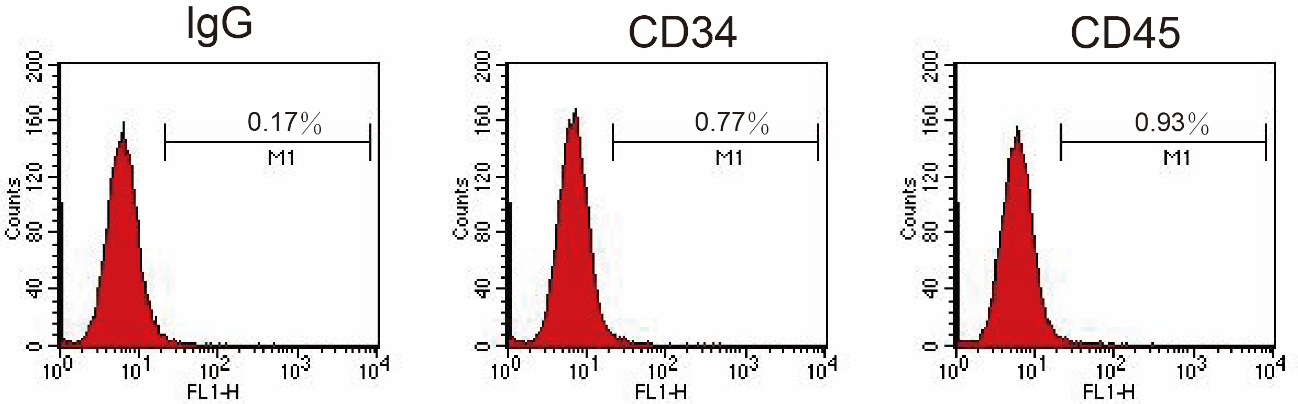


**Figure S3. Flow cytometry showed that no hematopoietic stem cell contamination in AnSCs.** The AnSCs were identified via flow cytometry with CD34 and CD45.

**Table S1. Summaries of antibodies used in the study**

| **Antibody** | **Host species** | **Manufacturer** | **Product code** | **Application** | **Dilution** |
| --- | --- | --- | --- | --- | --- |
| Anti-hTERT | Rabbit | Abcam | ab32020 | WB^1^ | 1:1000 |
| Anti-β-actin | Rabbit | CST | 8457 | WB | 1:1000 |
| Anti-CD44 | Rabbit | Proteintech | 15675-1 | IF^2^, Flow^3^ | 1:200 |
| Anti-CD90 | Rabbit | Bioss | bs-0778R | IF, Flow | 1:200 |
| Anti-CD34 | Rabbit | Beyotime | AF1387 | Flow | 1:100 |
| Anti-CD45 | Rabbit | Beyotime | AF7839 | Flow | 1:100 |
| Anti-CD81 | Rabbit | Abcam | ab109201 | WB | 1:1000 |
| Anti-ALIX | Rabbit | Abcam | ab275377 | WB | 1:1000 |
| Anti-TSG101 | Mouse | Abcam | ab83 | WB | 1:1000 |
| Anti-GM130 | Rabbit | Abcam | Ab52649 | WB | 1:1000 |
| IgG-Isotype control | Rabbit | Abcam | ab172730 | IF, Flow | 1:1000 |
| Donkey HRP-conjugated anti-IgG | Rabbit | Amersham | NA943 | WB | 1:5000 |
| Goat HRP-conjugated anti-IgG | Mouse | Amersham | NA931 | WB | 1:5000 |
| Goat Anti-IgG (H+L) Fluor488-conjugated | Rabbit | Affinity | S0018 | IF, Flow | 1:200 |

^1^WB: Western blot, ^2^IF: Immunofluorescence, ^3^Flow: Flow cytometry
